# Neuromechanical characteristics of breaststroke in adolescent swimmers across different performance levels: a comparative analysis

**DOI:** 10.3389/fbioe.2026.1870348

**Published:** 2026-06-10

**Authors:** Gongju Liu, Haoshui Zhu, Jing Ma, Houwei Zhu, Zhanyang He

**Affiliations:** 1 Key Laboratory of Aquatic Sports Science, General Administration of Sport, Zhejiang College of Sports, Hangzhou, China; 2 College of Physical Education and Health Sciences, Zhejiang Normal University, Jinhua, China; 3 School of Sport Science, Beijing Sport University, Beijing, China

**Keywords:** adolescents, breaststroke, muscle synergies, surface electromyography, swimming

## Abstract

**Background:**

Breaststroke is technically complex and requires high neuromuscular coordination. Because muscle synergies describe how multiple muscles are organized into low-dimensional activation modules, they provide a functional read-out of neuromuscular control development during adolescence. This study investigated neuromechanical characteristics of adolescent breaststroke swimmers across performance levels.

**Methods:**

Twenty-six male adolescent breaststroke swimmers were divided into elite (n = 13) and amateur (n = 13) groups. During five 25-m maximal breaststroke trials, two-dimensional underwater video and synchronized surface EMG from 16 muscles were collected. Main outcomes included discrete joint angles, the minimum number of synergies, variance accounted for (VAF), spatial weightings, and temporal activation coefficients. Group differences were assessed using independent-samples or Mann-Whitney U tests, FDR-adjusted spatial-weighting comparisons, SPM1D for activation curves, and Cohen’s d effect sizes.

**Results:**

At v2, the elite group showed greater hip flexion (43.78° ± 6.92° vs. 34.39° ± 11.32°, P = 0.018, d = 1.00), smaller knee flexion (122.93° ± 8.77° vs. 128.93° ± 4.84°, P = 0.041, d = −0.85), and smaller hip abduction (36.28° ± 7.19° vs. 45.22° ± 7.87°, P = 0.006, d = −1.19). VAF was similar between groups (91.27% ± 0.69% vs. 91.20% ± 0.73%, P = 0.583, d = 0.10), whereas the elite group required fewer minimum synergies (5.42 ± 0.72 vs. 6.03 ± 0.66, P < 0.001, d = −0.88). FDR-adjusted spatial-weighting and SPM1D analyses indicated that SYN2 and SYN4 were the main modules distinguishing the groups, with temporal differences concentrated from late glide to in-sweep.

**Conclusion:**

Performance-related differences mainly reflected lower-limb posture during glide and synergy control during propulsion-related phases. Fewer minimum synergies under comparable VAF conditions suggest greater synergy integration in high-level swimmers. These findings link adolescent breaststroke performance to task-specific multi-muscle coordination.

## 1 Introduction

Breaststroke is the most technically complex and coordination-demanding of the four competitive swimming strokes. Its propulsion is generated primarily by discontinuous and symmetrical underwater arm pulling and leg kick-squeeze actions, a movement pattern that produces substantial intra-cyclic velocity variation (IVV) of the body center of mass and considerable form drag throughout the stroke cycle (Barbosa et al., 2010; Colman et al., 1998; Gourgoulis et al., 2018). As a consequence, breaststroke is the slowest of the four competitive strokes. High-level breaststroke performance depends not only on optimized external limb kinematics, but also, to a large extent, on the precise spatiotemporal regulation of upper- and lower-limb coordination by the central nervous system (CNS) within very short time windows (Chollet et al., 2004; Seifert et al., 2014). Adolescence is a critical period for synaptic pruning and myelination within the CNS. Also, it represents a key window for the development of neuromuscular control and the consolidation of motor skills (Gogtay et al., 2004; Lloyd and Oliver, 2012). In practice, however, training in this age group often relies heavily on coaches’ subjective observations and externally oriented instruction, making it difficult to identify the internal neuromuscular coordination patterns associated with swimming performance. As a result, many adolescents with an initial technical foundation may become trapped in rigid movement patterns and compensatory force-production strategies as they attempt to progress toward higher performance levels.

Previous studies on breaststroke performance have mainly focused on macroscopic kinematic and kinetic variables, using methods such as three-dimensional motion capture and tethered force measurement systems to assess stroke kinematics and mechanical efficiency (Gao et al., 2025; Leblanc et al., 2005; Morouço et al., 2011; Takagi et al., 2004). However, these approaches largely treat the human body as a biomechanical output system and primarily quantify the external manifestations of the interaction between the swimmer and the aquatic environment. Although such methods effectively characterize movement outcomes, they provide limited insight into the underlying neuromuscular coordination patterns and muscle activation dynamics associated with movement generation. To investigate internal muscle activation during the underwater phase of swimming, surface electromyography (sEMG) has gradually been introduced into this field. Nevertheless, early EMG studies mostly relied on qualitative descriptions or isolated analyses of local electrophysiological features, such as root mean square amplitude, which not only overlooked the complex dynamic coupling among multiple muscles but also made quantitative comparisons across individuals difficult (Olstad et al., 2017). In addition, although the leg kick-squeeze plays a decisive role in breaststroke propulsion, quantitative EMG studies of lower-limb activation patterns, particularly those addressing integrated upper- and lower-limb coordination, remain scarce.

To overcome the limitations of isolated muscle analyses and to provide a more integrative understanding of neuromuscular coordination, muscle synergy theory has been increasingly applied in the field of motor control. This framework proposes that the CNS does not control each muscle independently, but rather recruits predefined low-dimensional modules (synergies) to activate groups of muscles simultaneously, thereby efficiently resolving the redundancy problem inherent in multi-joint movement (Bizzi and Cheung, 2013; He et al., 2025; Ting and Macpherson, 2005). Through non-negative matrix factorization (NMF), redundant sEMG signals can be decomposed into spatial structures and temporal activation coefficients, thereby enabling the identification of latent neural control strategies and the quantification of complex neuromuscular coordination patterns (Torres-Oviedo and Ting, 2007; Tresch et al., 2006). Muscle synergy theory has already been shown to provide important insights into control strategies in terrestrial movements and in continuous aquatic tasks such as freestyle swimming (Jie et al., 2024; Matsuura et al., 2020). However, for breaststroke, which is characterized by a highly complex technical structure and markedly discontinuous movement rhythm, the underlying synergy control mechanisms remain insufficiently understood. Existing evidence suggests that, in relatively simple motor tasks, differences in performance level do not necessarily alter the spatial structure of underlying muscle synergies. In contrast, when dealing with the complex and dynamically changing hydrodynamic constraints of breaststroke, the CNS may exhibit more pronounced task-specific regulation. Vaz et al. (2016), in a quantitative study of adult breaststroke swimmers, reported that although elite swimmers and beginners showed high similarity in synergy spatial structure, they differed substantially in temporal activation characteristics. Specifically, beginners exhibited significantly earlier activation of key propulsive muscles, such as the pectoralis major and rectus femoris, and the core synergy responsible for overall arm-leg coordination was also activated earlier (Vaz et al., 2016). Such temporal shifts have been considered an important mechanism underlying reduced underwater coordination efficiency in beginners. However, how this performance-level-dependent reorganization of synergy timing manifests in adolescents undergoing rapid neural development and motor skill consolidation has not yet been systematically investigated. Of particular interest is whether the developmental plasticity unique to adolescence leads to muscle synergy network characteristics that differ from those observed in adult swimmers.

Therefore, the present study aimed to investigate the neuromechanical characteristics of breaststroke in adolescent swimmers of different performance levels.We hypothesized that between-group differences would be more evident in the temporal activation patterns of muscle synergies and in external kinematic coordination, while possible task-specific differences in spatial muscle weightings were also explored.

## 2 Methods

### 2.1 Participants

Participants were recruited in May 2025 from the swimming teams of Zhejiang Normal University and Zhejiang Sports Vocational and Technical College through poster advertisements and social media announcements. All potential participants were required to meet the following general inclusion criteria: (1) healthy male adolescents aged 12–14 years; (2) no history of neurological disease or musculoskeletal injury; and (3) no joint injury or localized pain within the previous 6 months.

According to the study design, participants were further screened and assigned to one of two groups. The elite group was required to have more than 5 years of systematic competitive swimming training, to be engaged in regular high-intensity water-based training at the time of testing (mean frequency: six sessions per week, 2 h per session), and to have achieved a defined competitive standard, including participation in national youth championships. The amateur group was required to have approximately 3 years of recreational swimming or club-based training, to possess a basic level of breaststroke proficiency, and to be able to complete a 25-m maximal breaststroke trial in a continuous and technically acceptable manner.

After screening, a total of 26 participants were included in the study, comprising 13 elite swimmers and 13 amateur swimmers. Participant characteristics are presented in Table 1. To minimize the potential influence of fatigue accumulation and external factors on the test results, pre-test controls were implemented. Participants were instructed to refrain from any swimming competitions or high-intensity testing during the week before testing. During the 24 h preceding the experiment, they were required to obtain sufficient sleep, avoid strenuous physical activity, and refrain from consuming caffeine or other stimulant-containing beverages. The study protocol was reviewed and approved by the Ethics Committee of Zhejiang Normal University (Approval No. ZSRT2024283). All procedures were conducted in accordance with the Declaration of Helsinki. Because all participants were minors, the study procedures, aims, and potential risks were explained in detail to both the participants and their legal guardians before data collection. Written informed consent was obtained from all participants and their legal guardians before participation.

**TABLE 1:** Participant characteristics.

Group	n	Age (years)	Height (m)	Body mass (kg)	Training years (years)
Elite	13	13.6 ± 0.73	1.68 ± 0.021	52.3 ± 2.10	7.2 ± 1.25
Amateur	13	13.2 ± 0.54	1.61 ± 0.019	53.4 ± 1.70	2.3 ± 0.82

### 2.2 Experimental procedure and data acquisition

**FIGURE 1:**
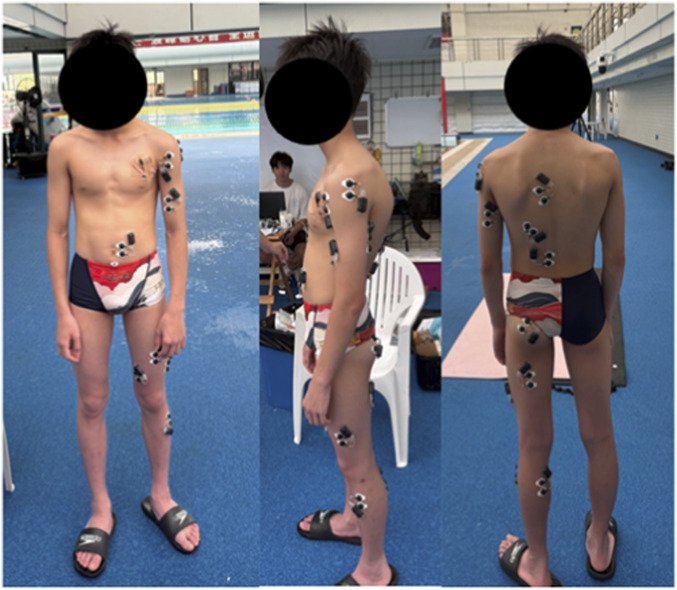
Placement of the surface EMG sensors.

All tests were conducted in a standard 25-m short-course pool. Before testing, the participants were informed about the experimental procedures and precautions for underwater testing. They then completed a 15-min swimming-specific warm-up on land and in water (Kilduff et al., 2011). After the warm-up, skin preparation was performed over the muscle bellies of 16 target muscles on the dominant side. The preparation procedure included shaving local body hair, lightly abrading the skin with fine sandpaper, and cleaning the skin with 75% alcohol swabs. After the skin had dried completely, surface EMG sensors (2000 Hz, MiniX Waterproof, Cometa) were placed along the direction of the muscle fibers. The 16 recorded muscles were the pectoralis major (PM), latissimus dorsi (LD), triceps brachii (TB), biceps brachii (BB), anterior deltoid (AD), posterior deltoid (PD), lower trapezius (LT), rectus abdominis (RA), erector spinae (ES), gluteus maximus (GMAX), rectus femoris (RF), biceps femoris (BF), vastus medialis (VM), vastus lateralis (VL), medial gastrocnemius (MG), and tibialis anterior (TA). To reduce the influence of the aquatic environment on electrode fixation and signal acquisition, all sensors were covered with a double layer of 3M Tegaderm transparent waterproof film (Figure 1).

After the participants entered the water wearing the sensors, the baseline signals of all 16 EMG channels were monitored using the acquisition software to confirm normal signal connection and the absence of obvious power-line interference. To synchronize the kinematic images and surface EMG signals, a visual-electrical synchronization procedure was used. At the beginning of formal data collection, three action cameras (120 Hz, GoPro HERO11 Black, GoPro) recorded the instant at which the LED indicator on the EMG sensor illuminated, and this instant was defined as the common time zero for synchronization of the two systems. Thereafter, the participants performed one to two trials of low-to-moderate intensity swimming in water to adapt to the sensors and waterproof covering. Following synchronization, the three GoPro cameras were arranged at different locations around the pool. One camera was positioned on the pool wall opposite the starting side to record frontal-plane kinematic data, while the other two cameras were positioned 15 m and 20 m from the pool wall to record sagittal-plane kinematic data.

After the adaptation phase, formal testing began from the lane start position using a push-off start from within the water. The testing task consisted of a 25-m maximal-effort breaststroke sprint performed at 100% relative intensity. Each participant completed five 25-m breaststroke trials. For each trial, two complete stroke cycles with the shortest cycle duration and clear video-EMG synchronization were selected for analysis, resulting in 10 cycles per participant. Cycle selection was performed by frame-by-frame inspection using Dartfish and synchronized surface-camera recordings. A 5-min rest interval was provided between successive trials to reduce the influence of repeated sprint efforts on subsequent performance.

### 2.3 Data processing

Based on previously reported methods for dividing the upper-limb phases of breaststroke (Gourgoulis and Nikodelis, 2022), and through frame-by-frame analysis of synchronized underwater video recordings, five key events (v1–v5) were identified. These events were used to divide a complete breaststroke cycle into four consecutive phases: recovery phase (v1–v2), glide phase (v2–v3), out-sweep phase (v3–v4), and in-sweep phase (v4–v5). Specifically, v1 was defined as the instant of maximal flexion of the right elbow and was taken as the start of the cycle (0%); v2 as the instant when the right elbow completed extension; v3 as the instant when the right hand began to move laterally outward; v4 as the instant of maximal lateral displacement of the right hand; and v5 as the instant when the right elbow again reached maximal flexion, which was also defined as the start of the subsequent cycle (100%). Accordingly, the recovery phase was defined from v1 to v2, the glide phase from v2 to v3, the out-sweep phase from v3 to v4, and the in-sweep phase from v4 to v5.

#### 2.3.1 Kinematic data extraction

Two-dimensional kinematic analysis of the synchronized underwater video recordings was performed using Dartfish video analysis software (Dartfish, Switzerland). Discrete joint angle variables were extracted at the key events defined in Section 2.3 (v1, v2, and v4). Kinematic data were obtained from both frontal-plane and sagittal-plane video recordings. To reduce measurement error, the cameras were fixed at predetermined positions and aligned as closely as possible with the frontal and sagittal movement planes. Joint angles were extracted frame by frame in Dartfish by identifying anatomical landmarks at the predefined key events. Only cycles in which the relevant body segments were clearly visible and the swimmer remained within the calibrated field of view were included. Because underwater refraction and parallax cannot be completely eliminated in two-dimensional video analysis, the kinematic results were interpreted as discrete two-dimensional joint-angle estimates rather than full three-dimensional movement descriptors.

From the frontal-plane recordings, upper-limb joint angle variables were extracted at v1 and v4, including shoulder abduction angle and elbow flexion angle. Here, v1 corresponded to the instant of maximal right elbow flexion and represented the start of the movement cycle, whereas v4 corresponded to the instant of maximal lateral displacement of the right hand and represented the end of the out-sweep phase. These upper-limb joint angles were used to characterize joint kinematics at the beginning of the cycle and at the end of the out-sweep phase.

From the sagittal-plane recordings, lower-limb joint angle variables were extracted at v2, including hip flexion angle, knee flexion angle, and ankle dorsiflexion/plantarflexion angle. The v2 event was defined as the instant at which the right elbow completed extension, corresponding to the transition between the end of recovery and the beginning of glide. These variables were used to characterize lower-limb joint kinematics in the sagittal plane at this transition point.

#### 2.3.2 EMG processing

The raw underwater surface EMG signals were processed in Python 3.11. First, based on the key events identified from the synchronized video recordings, 10 stable complete breaststroke cycles were extracted for each participant, with two cycles selected from each of the five 25-m trials. A stable cycle was defined as a complete v1-to-subsequent-v1 cycle in which all key events were clearly identifiable, the swimmer maintained continuous breaststroke without interruption or obvious technical disturbance, and the EMG signals showed no obvious motion artifact, signal dropout, or electrode detachment. When more than two cycles met these criteria within a trial, the two cycles with the shortest cycle duration were retained. The 10 selected cycles were subsequently time-normalized and averaged within each participant before group-level analysis to reduce the influence of within-subject cycle-to-cycle variability. The raw EMG signals were then preprocessed. To suppress power-line interference, a 50-Hz notch filter was first applied. Subsequently, a fourth-order zero-phase bidirectional Butterworth band-pass filter (20–400 Hz) was used to remove low-frequency motion artifacts and high-frequency noise. The filtered EMG signals were full-wave rectified and then smoothed using a fourth-order zero-phase bidirectional Butterworth low-pass filter with a cutoff frequency of 10 Hz to obtain the linear envelope.

A dynamic peak normalization procedure was used. This normalization approach was selected because MVIC testing is difficult to standardize in an aquatic environment, whereas dynamic peak normalization provides a task-specific reference value for comparing activation patterns during breaststroke. However, this approach may limit direct interpretation of absolute activation amplitude across participants. Specifically, the maximum linear envelope value observed for each target muscle across the 10 complete breaststroke cycles was taken as the normalization reference, and the envelope of each muscle was scaled to a dimensionless range of 0–1. To reduce the influence of inter-subject and inter-cycle differences in movement duration on temporal comparisons, the normalized EMG linear envelopes were time-normalized using cubic spline interpolation (He, 2024). Each complete breaststroke cycle (from v1 to the subsequent v1) was resampled to 404 data points, with the recovery, glide, out-sweep, and in-sweep phases each normalized to 101 data points. Finally, the EMG envelopes across the 10 normalized cycles were averaged for each participant to construct a non-negative data matrix V of size m×t , where m=16 denotes the number of muscles and t=404 denotes the number of time points per cycle. This matrix served as the input for subsequent non-negative matrix factorization (NMF). Muscle synergies were identified based on the estimated number of synergies and the corresponding muscle weightings. A muscle synergy was defined as active when its activation coefficient exceeded 30% (Xiong et al., 2018).

##### 2.3.2.1 Muscle synergy extraction and determination of the number of synergies

NMF was used to extract muscle synergy characteristics during breaststroke. For each participant, the preprocessed EMG matrix V was decomposed into the product of a spatial weighting matrix W and a temporal activation coefficient matrix H , such that (Equation 1)

$$V\approx \hat{V}=W\times H$$ (1)

where $V\in R_{+}^{m\times t}$ is the original non-negative EMG data matrix, $\hat{V}\in R_{+}^{m\times t}$ is the reconstructed matrix, $W\in R_{+}^{m\times n}$ is the spatial weighting matrix, $H\in R_{+}^{n\times t}$ is the temporal activation coefficient matrix and n denotes the number of synergies. The spatial weighting matrix W reflects the relative contribution of each muscle to each synergy, whereas the temporal activation coefficient matrix H describes the time-varying activation pattern of each synergy across the complete breaststroke cycle. The residual error matrix was defined as E = V − V̂.

The NMF solution was obtained by minimizing the reconstruction error between the original and reconstructed matrices (Equation 2):

$$\min_{W,H}\;{\left\|V-WH\right\|}_{F}^{2}$$ (2)

where $∥\cdot {∥}_{F}$ denotes the Frobenius norm. A multiplicative update algorithm was used for iterative optimization. The number of synergies n was searched from 1 to 16. For each value of n , W and H were randomly initialized 20 times, with a maximum of 1000 iterations, and the solution with the smallest reconstruction error was retained as the final result for that value of n .

To evaluate the reconstruction quality of the NMF model and determine the optimal number of synergies, the variance accounted for (VAF) was used as the criterion. Global VAF was calculated as follows Equation 3:

$$VAF_{\text{global}\;}=1-\frac{\sum_{i=1}^{m}\;\sum_{j=1}^{t}\;{\left(V_{ij}-{\hat{V}}_{ij}\right)}^{2}}{\sum_{i=1}^{m}\;\sum_{j=1}^{t}\;V_{ij}^{2}}$$ (3)

where $V_{ij}$ is the value of the original EMG matrix for muscle i at time point j , and ${\hat{V}}_{ij}$ is the corresponding reconstructed value. Global VAF represents the proportion of the overall variance in the original EMG signal explained by the extracted synergies. The minimum number of synergies n at which global VAF first reached or exceeded 90% was defined as the optimal number of synergies required for each participant to perform the breaststroke task. This criterion was used to control model complexity while maintaining adequate reconstruction accuracy.

##### 2.3.2.2 Synergy matching and clustering analysis

K-means clustering was used to match the extracted synergies across participants. All spatial weighting vectors $W_{i}$ obtained from all participants were pooled into a feature set, and the column vectors of the spatial weighting matrix were used as clustering inputs. The number of clusters k was set to the most representative maximum number of synergies observed across participants; in the present study, k=6 . The clustering procedure was repeated with different random initial centroids, and the final matching was checked using cosine similarity to ensure that synergies assigned to the same cluster showed consistent spatial weighting patterns. At the initial stage, six weighting vectors were randomly selected from the feature set as the initial cluster centroids, $C_{1},\ldots ,C_{6}$ .

To eliminate the influence of differences in absolute vector magnitude, cosine similarity was used to quantify the similarity between synergy weighting patterns. For the i -th synergy vector $W_{i}$ and the j -th cluster centroid $C_{j}$ , cosine similarity was defined as Equation 4

$$\text{Similarity}\left(W_{i},C_{j}\right)=\frac{W_{i}\cdot C_{j}}{\left\|W_{i}\right\|\left\|C_{j}\right\|}$$ (4)

Because 16 target muscles were recorded in the present study, this expression can be expanded as Equation 5

$$\text{Similarity}\left(W_{i},C_{j}\right)=\frac{\sum_{m=1}^{16}\;W_{i,m}C_{j,m}}{\sqrt{\sum_{m=1}^{16}\;W_{i,m}^{2}}\sqrt{\sum_{m=1}^{16}\;C_{j,m}^{2}}}$$ (5)

At each iteration, each synergy vector $W_{i}$ was assigned to the cluster whose centroid yielded the highest cosine similarity. After all vectors had been assigned, the centroid of each cluster was updated by taking the arithmetic mean of the weighting vectors within that cluster (Equation 6):

$$C_{j}^{\text{new}\;}=\frac{1}{N_{j}}\sum_{W_{i}\in S_{j}}\;W_{i}$$ (6)

where $S_{j}$ denotes the j -th cluster and $N_{j}$ denotes the number of synergy vectors assigned to that cluster. The assignment and update steps were repeated until the centroids no longer changed or the preset maximum number of iterations was reached. Ultimately, each cluster represented a group-level synergy pattern used for subsequent between-group comparisons.

### 2.4 Statistical analysis

Statistical analyses were performed using MATLAB R2024b for SPM analysis and SPSS 26.0 (IBM Corp., Armonk, NY, USA) for conventional statistical testing.

For discrete variables, including kinematic parameters, number of synergies, spatial weights of each fundamental synergy, and activation timing characteristics, data normality was assessed using the Shapiro–Wilk test and homogeneity of variance was assessed using Levene’s test. Variables satisfying the assumptions of normality and homogeneity of variance were compared between the elite and amateur groups using independent-samples t-tests. Variables that did not satisfy the normality assumption were analyzed using the Mann–Whitney U test. For the muscle-by-synergy comparisons of spatial weightings, P values were adjusted within each synergy using the Benjamini–Hochberg false discovery rate (FDR) procedure across the 16 muscle comparisons. Statistical significance for these comparisons was accepted at adjusted P < 0.05. Cohen’s d effect sizes were calculated for the main group comparisons, with positive values indicating greater values in the elite group and negative values indicating greater values in the amateur group.

For continuous time-series variables, namely, the activation coefficient curves of the muscle synergies, one-dimensional statistical parametric mapping (SPM1D) based on random field theory was used to perform independent-samples t-tests, in order to compare temporal differences between groups across the entire normalized stroke cycle (Pataky et al., 2013). A significant between-group difference was identified when the SPM $\left\{t\right\}$ trajectory exceeded the critical threshold over a continuous time interval. All tests were two-sided, with α set at 0.05.

## 3 Results

### 3.1 Kinematic

The comparison of joint kinematic parameters between adolescent breaststroke swimmers of different performance levels is presented in Table 2. Significant between-group differences were observed in lower-limb joint angle variables at v2. Specifically, the elite group showed a significantly greater hip flexion angle than the amateur group (43.78° ± 6.92° vs. 34.39° ± 11.32°, P = 0.018, Cohen’s d = 1.00), whereas the knee flexion angle was significantly smaller in the elite group than in the amateur group (122.93° ± 8.77° vs. 128.93° ± 4.84°, P = 0.041, Cohen’s d = −0.85). In addition, the elite group exhibited a significantly smaller hip abduction angle than the amateur group (36.28° ± 7.19° vs. 45.22° ± 7.87°, P = 0.006, Cohen’s d = −1.19).

**TABLE 2:** Comparison of joint kinematic characteristics during breaststroke between adolescent swimmers of different performance levels.

Time	Joint	Elite group (°)	Amateur group (°)	P Value	Cohen’s d
v1	Shoulder abduction angle	139.32 ± 7.20	137.95 ± 13.75	0.755	0.12
	Elbow flexion angle	105.50 ± 5.59	106.75 ± 11.10	0.721	−0.14
v4	Shoulder abduction angle	170.32 ± 7.49	165.14 ± 12.85	0.463	0.49
	Elbow flexion angle	133.45 ± 11.28	135.80 ± 15.50	0.671	−0.17
v2	Hip flexion angle	43.78 ± 6.92	34.39 ± 11.32	0.018	1.00
	Knee flexion angle	122.93 ± 8.77	128.93 ± 4.84	0.041	−0.85
	Ankle dorsiflexion/plantarflexion angle	0.30 ± 10.36	−4.18 ± 8.63	0.383	0.47
	Hip abduction angle	36.28 ± 7.19	45.22 ± 7.87	0.006	−1.19

### 3.2 Muscle synergies

#### 3.2.1 Minimum number of muscle synergies and variance accounted for

As shown in Table 3, when the global variance accounted for (VAF) first reached the predefined threshold of 90%, the corresponding global VAF values were 91.27% ± 0.69% in the elite group and 91.20% ± 0.73% in the amateur group, with no significant between-group difference (t = 0.55, P = 0.583, Cohen’s d = 0.10). However, the minimum number of muscle synergies required to achieve this level of reconstruction accuracy differed significantly between groups. The elite group required significantly fewer synergies than the amateur group (5.42 ± 0.72 vs. 6.03 ± 0.66, t = −5.05, P < 0.001, Cohen’s d = −0.88).

**TABLE 3:** Comparison of the minimum number of muscle synergies and VAF between adolescent swimmers of different performance levels.

Variable	Elite group	Amateur group	t value	P Value
Minimum number of synergies	5.42 ± 0.72	6.03 ± 0.66	−5.05	<0.001
VAF (%)	91.27 ± 0.69	91.20 ± 0.73	0.55	0.583

#### 3.2.2 Spatial weightings and temporal activation characteristics of the synergies

In SYN1, the elite group showed significantly higher relative weightings of LT and RF than the amateur group (adjusted P < 0.001 and adjusted P = 0.035, respectively), whereas the amateur group showed significantly higher relative weightings of ES, LD, BF, and GMAX than the elite group (adjusted P < 0.001, adjusted P = 0.044, adjusted P = 0.002, and adjusted P < 0.001, respectively). No significant between-group differences were observed for the remaining muscles (Table 4).

**TABLE 4:** Comparison of muscle activation weightings across the synergies.

Muscle	SYN1 elite group	SYN1 amateur group	adj. P	SYN2 elite group	SYN2 amateur group	adj. P	SYN3 elite group	SYN3 amateur group	adj. P
TB	0.042 ± 0.057	0.033 ± 0.057	0.660	0.102 ± 0.114	0.037 ± 0.056	<0.001	0.146 ± 0.155	0.141 ± 0.167	0.850
BB	0.127 ± 0.154	0.068 ± 0.127	0.248	0.028 ± 0.053	0.121 ± 0.074	<0.001	0.483 ± 0.217	0.491 ± 0.212	0.850
AD	0.109 ± 0.130	0.103 ± 0.160	0.914	0.137 ± 0.120	0.281 ± 0.122	<0.001	0.137 ± 0.184	0.014 ± 0.059	<0.001
PD	0.012 ± 0.026	0.040 ± 0.078	0.293	0.306 ± 0.110	0.064 ± 0.063	<0.001	0.047 ± 0.071	0.039 ± 0.078	0.715
ES	0.153 ± 0.224	0.612 ± 0.202	<0.001	0.030 ± 0.045	0.030 ± 0.061	0.964	0.204 ± 0.184	0.195 ± 0.180	0.850
LD	0.024 ± 0.026	0.098 ± 0.107	0.044	0.010 ± 0.014	0.013 ± 0.029	0.619	0.337 ± 0.229	0.450 ± 0.210	0.018
RA	0.045 ± 0.101	0.010 ± 0.027	0.081	0.045 ± 0.064	0.254 ± 0.164	<0.001	0.103 ± 0.140	0.068 ± 0.100	0.191
LT	0.854 ± 0.158	0.177 ± 0.146	<0.001	0.204 ± 0.139	0.117 ± 0.121	<0.001	0.075 ± 0.092	0.100 ± 0.118	0.246
PM	0.029 ± 0.054	0.134 ± 0.187	0.090	0.002 ± 0.009	0.010 ± 0.028	0.056	0.518 ± 0.187	0.461 ± 0.194	0.191
MG	0.035 ± 0.050	0.075 ± 0.121	0.307	0.027 ± 0.053	0.040 ± 0.052	0.193	0.056 ± 0.063	0.101 ± 0.141	0.080
TA	0.179 ± 0.159	0.080 ± 0.129	0.057	0.280 ± 0.116	0.356 ± 0.115	<0.001	0.003 ± 0.010	0.012 ± 0.035	0.154
BF	0.043 ± 0.086	0.259 ± 0.207	0.002	0.234 ± 0.111	0.053 ± 0.089	<0.001	0.021 ± 0.055	0.041 ± 0.086	0.191
RF	0.047 ± 0.086	0.006 ± 0.030	0.035	0.389 ± 0.075	0.409 ± 0.100	0.263	0.006 ± 0.012	0.003 ± 0.005	0.191
VM	0.027 ± 0.039	0.013 ± 0.045	0.354	0.405 ± 0.060	0.433 ± 0.079	0.043	0.005 ± 0.018	0.004 ± 0.006	0.636
VL	0.012 ± 0.032	0.002 ± 0.006	0.093	0.419 ± 0.067	0.433 ± 0.071	0.300	0.006 ± 0.011	0.004 ± 0.008	0.246
GMAX	0.075 ± 0.083	0.405 ± 0.275	<0.001	0.295 ± 0.113	0.119 ± 0.134	<0.001	0.085 ± 0.104	0.081 ± 0.074	0.850

Muscle	SYN4 elite group	SYN4 amateur group	adj. P	SYN5 elite group	SYN5 amateur group	adj. P	SYN6 elite group	SYN6 amateur group	adj. P
TB	0.640 ± 0.191	0.607 ± 0.168	0.516	0.224 ± 0.197	0.048 ± 0.055	<0.001	0.096 ± 0.112	0.019 ± 0.053	<0.001
BB	0.060 ± 0.083	0.053 ± 0.108	0.794	0.056 ± 0.135	0.040 ± 0.067	0.610	0.015 ± 0.029	0.169 ± 0.111	<0.001
AD	0.032 ± 0.075	0.008 ± 0.022	0.043	0.681 ± 0.208	0.178 ± 0.140	<0.001	0.217 ± 0.170	0.414 ± 0.181	<0.001
PD	0.031 ± 0.056	0.619 ± 0.206	<0.001	0.060 ± 0.088	0.005 ± 0.016	<0.001	0.359 ± 0.179	0.197 ± 0.110	<0.001
ES	0.115 ± 0.144	0.035 ± 0.064	<0.001	0.124 ± 0.175	0.167 ± 0.132	0.307	0.275 ± 0.181	0.185 ± 0.163	0.033
LD	0.311 ± 0.203	0.134 ± 0.134	<0.001	0.025 ± 0.045	0.017 ± 0.031	0.467	0.028 ± 0.045	0.016 ± 0.028	0.183
RA	0.288 ± 0.320	0.104 ± 0.107	<0.001	0.260 ± 0.197	0.242 ± 0.196	0.701	0.064 ± 0.092	0.054 ± 0.094	0.626
LT	0.042 ± 0.064	0.155 ± 0.112	<0.001	0.163 ± 0.171	0.071 ± 0.153	0.016	0.193 ± 0.144	0.452 ± 0.186	<0.001
PM	0.206 ± 0.209	0.126 ± 0.133	0.048	0.019 ± 0.037	0.015 ± 0.022	0.669	0.003 ± 0.011	0.006 ± 0.016	0.466
MG	0.081 ± 0.071	0.078 ± 0.145	0.889	0.024 ± 0.046	0.063 ± 0.108	0.030	0.546 ± 0.134	0.479 ± 0.208	0.110
TA	0.055 ± 0.065	0.012 ± 0.047	<0.001	0.295 ± 0.207	0.624 ± 0.161	<0.001	0.075 ± 0.123	0.007 ± 0.018	0.002
BF	0.017 ± 0.024	0.007 ± 0.029	0.155	0.095 ± 0.129	0.368 ± 0.240	<0.001	0.369 ± 0.154	0.112 ± 0.148	<0.001
RF	0.010 ± 0.021	0.011 ± 0.016	0.794	0.014 ± 0.033	0.019 ± 0.048	0.610	0.007 ± 0.019	0.071 ± 0.123	0.002
VM	0.010 ± 0.027	0.006 ± 0.012	0.477	0.011 ± 0.020	0.014 ± 0.044	0.709	0.117 ± 0.110	0.093 ± 0.095	0.320
VL	0.015 ± 0.020	0.017 ± 0.020	0.765	0.005 ± 0.013	0.021 ± 0.058	0.085	0.059 ± 0.077	0.143 ± 0.100	<0.001
GMAX	0.199 ± 0.179	0.018 ± 0.029	<0.001	0.035 ± 0.049	0.314 ± 0.200	<0.001	0.135 ± 0.114	0.086 ± 0.112	0.075

adj. P values were obtained using the Benjamini–Hochberg false discovery rate correction within each synergy.

In SYN2, the elite group showed significantly higher relative weightings of TB, PD, LT, BF, and GMAX than the amateur group (all adjusted P < 0.001), whereas the amateur group showed significantly higher relative weightings of BB, AD, RA, TA, and VM than the elite group (adjusted P < 0.001, adjusted P < 0.001, adjusted P < 0.001, adjusted P < 0.001, and adjusted P = 0.043, respectively). No significant between-group differences were observed for the remaining muscles.

In SYN3, only AD showed a significantly higher relative weighting in the elite group than in the amateur group (adjusted P < 0.001), whereas LD showed a significantly higher relative weighting in the amateur group than in the elite group (adjusted P = 0.018). No significant between-group differences were observed for the remaining muscles.

In SYN4, the elite group showed significantly higher relative weightings of AD, ES, LD, RA, PM, TA, and GMAX than the amateur group (adjusted P = 0.043, adjusted P < 0.001, adjusted P < 0.001, adjusted P < 0.001, adjusted P = 0.048, adjusted P < 0.001, and adjusted P < 0.001, respectively), whereas the amateur group showed significantly higher relative weightings of PD and LT than the elite group (both adjusted P < 0.001). No significant between-group differences were observed for the remaining muscles.

In SYN5, the elite group showed significantly higher relative weightings of TB, AD, PD, and LT than the amateur group (adjusted P < 0.001, adjusted P < 0.001, adjusted P < 0.001, and adjusted P = 0.016, respectively), whereas the amateur group showed significantly higher relative weightings of MG, TA, BF, and GMAX than the elite group (adjusted P = 0.030, adjusted P < 0.001, adjusted P < 0.001, and adjusted P < 0.001, respectively). No significant between-group differences were observed for the remaining muscles.

**FIGURE 2:**
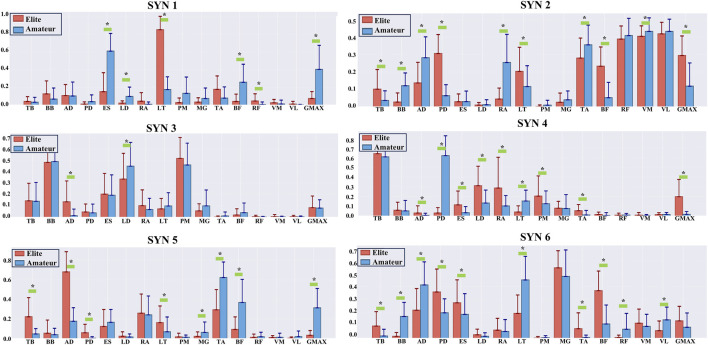
Muscle activation weightings of the synergies in each group. Note: Data are presented as mean ± SD, and error bars represent standard deviation. * indicates a significant between-group difference at adjusted P < 0.05 after Benjamini–Hochberg FDR correction. TB, triceps brachii; BB, biceps brachii; AD, anterior deltoid; PD, posterior deltoid; ES, erector spinae; LD, latissimus dorsi; RA, rectus abdominis; LT, lower trapezius; PM, pectoralis major; MG, medial gastrocnemius; TA, tibialis anterior; BF, biceps femoris; RF, rectus femoris; VM, vastus medialis; VL, vastus lateralis; GMAX, gluteus maximus.

In SYN6, the elite group showed significantly higher relative weightings of TB, PD, ES, TA, and BF than the amateur group (adjusted P < 0.001, adjusted P < 0.001, adjusted P = 0.033, adjusted P = 0.002, and adjusted P < 0.001, respectively), whereas the amateur group showed significantly higher relative weightings of BB, AD, LT, RF, and VL than the elite group (adjusted P < 0.001, adjusted P < 0.001, adjusted P < 0.001, adjusted P = 0.002, and adjusted P < 0.001, respectively). No significant between-group differences were observed for the remaining muscles (Figure 2). Cohen’s d effect sizes for the spatial weighting comparisons are provided in Supplementary Table 1.

**FIGURE 3:**
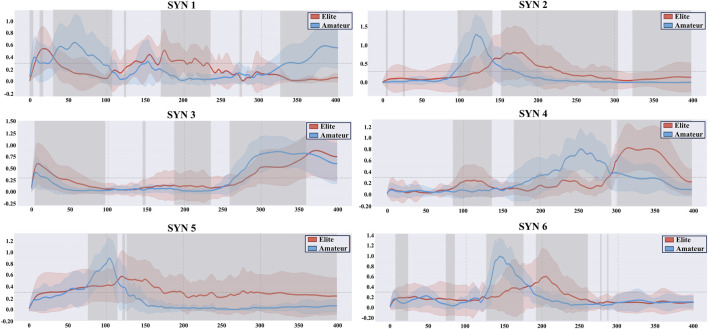
Activation coefficients of the synergies in each group.

Because each stroke cycle was normalized to 404 data points, the significant intervals are reported as normalized data-point indices from 0 to 403 rather than percentages. The SPM1d results showed significant between-group differences in the activation coefficient curves of all six fundamental synergies (all P < 0.05). Specifically, significant differences in SYN1 were observed over 0–5, 20–25, 35–104, 125–139, 173–238, 276–282, and 325–403 of the normalized cycle. Significant differences in SYN2 were observed over 23–28, 94–140, 153–306, and 326–403 of the normalized cycle. Significant differences in SYN3 were observed over 6–96, 187–234, and 264–355 of the normalized cycle. Significant differences in SYN4 were observed over 85–135, 176–293, and 301–403 of the normalized cycle. Significant differences in SYN5 were observed over 81–112 and 126–403 of the normalized cycle. Significant differences in SYN6 were observed over 12–23, 76–87, 129–175, 191–264, and 283–291 of the normalized cycle. No significant between-group differences were observed over the remaining portions of the normalized cycle (Figure 3; Table 5).

**TABLE 5:** Distribution of significant between-group intervals in the activation timing of muscle synergies.

Muscle synergy	Significant interval	P Value
SYN1	0–5	0.035
	20–25	0.049
	35–104	0.037
	125–139	0.049
	173–238	0.030
	276–282	0.022
	325–403	0.034
SYN2	23–28	0.049
	94–140	0.032
	153–306	0.048
	326–403	0.044
SYN3	6–96	0.037
	187–234	0.044
	264–355	0.041
SYN4	85–135	0.039
	176–293	0.050
	301–403	0.048
SYN5	81–112	0.034
	126–403	0.043
SYN6	12–23	0.050
	76–87	0.050
	129–175	0.044
	191–264	0.042
	283–291	0.048

## 4 Discussion

The present study aimed to investigate the neuromechanical characteristics of adolescent breaststroke swimmers of different performance levels by comparing the elite and amateur groups in terms of kinematics, muscle synergy structure, and temporal activation patterns across a complete breaststroke cycle. The main findings were as follows. First, the between-group kinematic differences were primarily observed at v2, with the elite group exhibiting a greater hip flexion angle, a smaller knee flexion angle, and a smaller hip abduction angle. Second, the elite group required significantly fewer minimum muscle synergies than the amateur group to complete a full breaststroke cycle. Third, the spatiotemporal neuromuscular differences between groups were mainly distributed from the late glide phase to the in-sweep phase, with SYN2 and SYN4 emerging as the principal synergies distinguishing adolescent swimmers of different performance levels, reflecting lower-limb-dominant multi-joint coordination and upper-limb propulsion with trunk coordination, respectively.

The present results indicate that the between-group kinematic differences in adolescent breaststroke swimmers were not distributed across the entire stroke cycle, but were mainly concentrated in lower-limb joint kinematics at v2. At least at the two discrete events examined in this study, v1 and v4, no between-group differences were observed in shoulder abduction or elbow flexion angles, suggesting a relatively high degree of consistency in discrete upper-limb joint configuration at these key arm-stroke instants. In light of previous findings that the arm pull and out-sweep trajectory in breaststroke is subject to relatively well-defined technical requirements (Maglischo, 2003), the absence of significant between-group differences in upper-limb kinematics at v1 and v4 may be expected. Accordingly, the external technical differences observed in the present study were primarily manifested in lower-limb posture organization at the transition from the end of arm recovery to the beginning of glide. In contrast, the elite group exhibited a greater hip flexion angle, a smaller knee flexion angle, and a smaller hip abduction angle at v2, indicating that the main between-group differences were concentrated in lower-limb spatial configuration at the onset of glide. In particular, the smaller hip abduction angle suggests that the lower limbs of the elite group were less laterally separated and more closely aligned with the body longitudinal axis at this instant. This finding is consistent with the report by Takagi et al. that high-level breaststroke swimmers tend to adopt a narrower leg recovery pattern to reduce drag (Takagi et al., 2004). From a biomechanical perspective, reduced lateral spreading of the lower limbs may help decrease the frontal area during glide, thereby reducing drag-related losses during the non-propulsive phase. At the same time, the combination of a greater hip flexion angle and a smaller knee flexion angle in the elite group suggests that their lower-limb configuration at this key instant was characterized not simply by a greater recovery amplitude, but by a distinct pattern of joint coordination compared with the amateur group. This finding implies that high-level adolescent breaststroke swimmers may adopt a preparatory lower-limb posture at the transition from arm extension to glide that may be associated with better drag control and preparation for subsequent propulsion. In addition, both groups exhibited relatively large within-group variability in ankle dorsiflexion/plantarflexion angle at v2, suggesting that this distal joint variable may be subject to considerable individual variability in adolescent breaststroke swimmers. This result is consistent with the findings of Leblanc et al., who reported inter-individual variation in arm-leg coordination timing among swimmers of different performance levels (Bizzi and Cheung, 2013). Therefore, compared with hip and knee configuration, the discrete ankle angle at this instant may be more strongly influenced by individual technical habits and movement rhythm. Taken together, the present findings indicate that the external kinematic differences between adolescent breaststroke swimmers of different performance levels are mainly reflected in lower-limb kinematics at v2, and that these differences may be associated with both drag control during glide and preparation for subsequent propulsion.

Both groups showed similar reconstruction accuracy when the global VAF first reached the predefined threshold of 90%, indicating that the NMF model adequately represented the modular muscle control characteristics of breaststroke (Bizzi and Cheung, 2013). Under this condition, however, the elite group required significantly fewer minimum synergies than the amateur group, suggesting a higher degree of synergy integration when performing the same task. According to Bernstein’s theory of motor degrees of freedom, one of the defining features of skilled motor control is the effective organization of a high-dimensional system (Ting et al., 2015). Therefore, a smaller minimum number of synergies does not imply that the movement is simpler; rather, it may reflect a more integrated modular organization that enables high-level swimmers to coordinate multiple muscles more efficiently. This interpretation is consistent with previous findings showing that training can modify the organization of synergy structure (Sawers et al., 2015), and further suggests that high-level adolescent breaststroke swimmers may be associated with greater technical economy (Frère and Hug, 2012).

Across all identified synergies, SYN2 and SYN4 showed more continuous and broader between-group temporal differences, indicating that neuromuscular differences between adolescent breaststroke swimmers of different performance levels were concentrated primarily in propulsion-related phases rather than being uniformly distributed throughout the entire stroke cycle. Specifically, the differences in SYN2 were mainly located from the late glide phase to the early in-sweep phase, whereas the differences in SYN4 were mainly distributed across the out-sweep and in-sweep phases. These findings suggest that the distinction between higher- and lower-level swimmers is reflected not only in propulsive output during propulsion, but also in the synergy organization associated with the preparation for and execution of propulsion.

SYN2 was primarily dominated by lower-limb muscles, including RF, VM, and VL, with additional contributions from BF, GMAX, and TA, reflecting a lower-limb-dominant multi-joint coordination pattern. Notably, the between-group differences in SYN2 first emerged during the late glide phase rather than being confined to the end of propulsion. This suggests that the difference between swimmers of different performance levels is not solely related to lower-limb force generation, but also to how the timing of lower-limb recruitment is coupled with upper-limb movement rhythm. This finding is consistent with the lower-limb joint configuration differences observed at v2 in the present study, where the elite group had already exhibited a lower-limb posture distinct from that of the amateur group at the transition from the end of arm recovery to the beginning of glide. Taken together, these results suggest that the technical advantage of high-level adolescent breaststroke swimmers may not derive solely from greater propulsive output, but also from a more orderly pattern of lower-limb synergy recruitment during the transition from glide to propulsion.

The between-group differences in SYN4 were mainly distributed across the out-sweep and in-sweep phases and were primarily dominated by TB, LD, RA, and PM, with additional involvement of some hip-related muscles. This weighting pattern suggests that SYN4 is associated not only with upper-limb propulsion, but also with trunk stabilization and intersegmental force transmission during propulsion (Torres-Oviedo and Ting, 2007). Compared with the amateur group, the elite group demonstrated greater involvement of trunk-related muscles in this module, indicating that their neuromuscular organization during propulsion was not limited to local upper-limb force generation, but was more likely characterized by an integrated pattern of upper-limb propulsion and trunk coordination. In contrast, the amateur group showed greater reliance on posterior shoulder-related muscles within this module, suggesting that their synergy organization during propulsion depended more heavily on local muscle recruitment. Overall, the findings for SYN4 indicate that between-group differences during propulsion involve not only upper-limb movement itself, but also the manner in which trunk coordination is organized.

Compared with SYN2 and SYN4, SYN1 and SYN3 reflected synergy characteristics more closely related to postural maintenance and upper-limb catch-related functions, respectively. In SYN1, the elite group showed higher weightings for LT and RF, whereas the amateur group showed higher weightings for ES, GMAX, BF, and LD, indicating different synergy organizations related to postural maintenance and shoulder-girdle or lower-limb stabilization between groups. Given the important role of the lower trapezius in scapular stabilization and upper-limb forward positioning (Hug et al., 2011), the greater contribution of LT in the elite group may reflect a different organizational strategy during streamline formation and body posture transition. In contrast, the greater involvement of posterior-chain muscles in the amateur group suggests a different muscle recruitment strategy for postural maintenance (Torres-Oviedo and Ting, 2007). In SYN3, the elite group showed a higher weighting of AD, whereas the amateur group showed a higher weighting of LD, suggesting that the two groups may have adopted different shoulder-control strategies during upper-limb forward positioning and catch preparation. Overall, the results for SYN1 and SYN3 indicate that, in addition to propulsion-related modules, adolescent breaststroke swimmers of different performance levels also differ in selected synergy weightings during postural control and upper-limb preparation phases.

For SYN5 and SYN6, the differences in SYN5 were mainly distributed from the late recovery phase to the early glide phase, whereas the differences in SYN6 were mainly distributed from the late glide phase to the early-to-mid out-sweep phase. These findings suggest that, beyond the core propulsion-related modules, adolescent breaststroke swimmers of different performance levels also exhibit localized differences in synergy modulation during the recovery-to-glide and glide-to-out-sweep transitions. Functionally, SYN5 and SYN6 do not appear to represent dominant modules of the complete propulsion process, but rather reflect the temporal linkage characteristics of movement transitions. Because breaststroke requires a high degree of spatiotemporal coordination between the upper and lower limbs (Seifert et al., 2014), between-group differences in these two synergies suggest that local muscle recruitment strategies during phase transitions also differ between swimmers of different performance levels. Thus, in addition to the core propulsion-related synergy modules, between-group differences in adolescent breaststroke swimmers are also reflected in local neuromuscular regulation during several transitional phases, although their explanatory importance appears to be less than that of SYN2 and SYN4.

This study has several limitations. First, the sample size was relatively small, which may have limited the statistical power and generalizability of the findings. Second, only male adolescent breaststroke swimmers were included; therefore, the conclusions are primarily applicable to male athletes and cannot be generalized to female swimmers (Shen et al., 2025). Future studies with larger samples, including swimmers of different sexes and broader age ranges, would help further verify and extend the present findings. Third, because only two-dimensional video analysis and discrete joint angles were used, the present kinematic data cannot fully characterize the three-dimensional nature of breaststroke or directly quantify drag, propulsive force, or swimming velocity fluctuations. Future studies incorporating three-dimensional motion analysis, hydrodynamic assessment, or synchronized velocity measurement would provide a more comprehensive understanding of the biomechanical mechanisms underlying performance-related differences. In addition, biological maturation status, pubertal stage, maturity offset, and additional anthropometric characteristics were not directly assessed. Although the chronological age range was restricted to 12–14 years, incorporating maturation-related indicators in future research would provide a more comprehensive understanding of neuromuscular control characteristics in adolescent swimmers.

## 5 Conclusion

1. Adolescent swimmers of different performance levels showed similar upper-limb kinematic parameters during the arm stroke, whereas their key technical difference was observed during the glide phase associated with arm–leg coordination.
2. The elite group required fewer minimum muscle synergies than the amateur group, indicating a higher degree of muscle synergy integration when performing the same technical task.
3. The spatiotemporal neuromuscular differences between adolescent breaststroke swimmers of different performance levels were mainly concentrated from the late glide phase to the in-sweep phase.
4. SYN2 and SYN4 were the principal synergy modules distinguishing adolescent breaststroke swimmers of different performance levels, representing lower-limb-dominant coordination during the glide phase and upper-limb propulsion with trunk coordination during the out-sweep and in-sweep phases, respectively.

## Data Availability

The datasets presented in this study can be found in online repositories. The names of the repository/repositories and accession number(s) can be found in the article/Supplementary Material.
