# Supplementary material for: Neuromechanical characteristics of breaststroke in adolescent swimmers across different performance levels: a comparative analysis
Source: Front Bioeng Biotechnol. 2026 Jun 10;14:1870348. doi: 10.3389/fbioe.2026.1870348 (PMC13291545; doi:10.3389/fbioe.2026.1870348)
Supplement: Supplementary file 1 [file Table1.docx]

**Supplementary Material**

**Supplementary Table 1. Cohen's d effect sizes for between-group comparisons of muscle activation weightings across synergies.**

| **Muscle** | **SYN1** | **SYN2** | **SYN3** | **SYN4** | **SYN5** | **SYN6** |
| --- | --- | --- | --- | --- | --- | --- |
| TB | 0.158 | 0.722 | 0.032 | 0.189 | 1.131 | 0.865 |
| BB | 0.440 | -1.467 | -0.038 | 0.072 | 0.144 | -1.938 |
| AD | 0.034 | -1.197 | 0.885 | 0.510 | 2.748 | -1.119 |
| PD | -0.393 | 2.674 | 0.103 | -3.442 | 0.806 | 1.076 |
| ES | -2.219 | -0.008 | 0.052 | 0.818 | -0.271 | 0.521 |
| LD | -0.784 | -0.099 | -0.518 | 1.106 | 0.206 | 0.323 |
| RA | 0.665 | -1.705 | 0.286 | 0.894 | 0.092 | 0.108 |
| LT | 4.558 | 0.669 | -0.239 | -1.154 | 0.567 | -1.564 |
| PM | -0.633 | -0.373 | 0.302 | 0.489 | 0.113 | -0.173 |
| MG | -0.368 | -0.262 | -0.411 | 0.030 | -0.511 | 0.389 |
| TA | 0.730 | -0.656 | -0.348 | 0.807 | -1.736 | 0.753 |
| BF | -1.157 | 1.797 | -0.280 | 0.365 | -1.497 | 1.699 |
| RF | 0.835 | -0.223 | 0.308 | -0.071 | -0.141 | -0.742 |
| VM | 0.324 | -0.399 | 0.130 | 0.214 | -0.077 | 0.241 |
| VL | 0.613 | -0.200 | 0.247 | -0.108 | -0.413 | -0.949 |
| GMAX | -1.345 | 1.424 | 0.041 | 1.703 | -2.094 | 0.437 |

Note. Values are Cohen's d effect sizes for the elite group relative to the amateur group. Positive values indicate higher relative muscle weighting in the elite group, whereas negative values indicate higher relative muscle weighting in the amateur group. SYN1-SYN6, muscle synergies 1-6; TB, triceps brachii; BB, biceps brachii; AD, anterior deltoid; PD, posterior deltoid; ES, erector spinae; LD, latissimus dorsi; RA, rectus abdominis; LT, lower trapezius; PM, pectoralis major; MG, medial gastrocnemius; TA, tibialis anterior; BF, biceps femoris; RF, rectus femoris; VM, vastus medialis; VL, vastus lateralis; GMAX, gluteus maximus.
